# Supplementary material for: Mapping the evolution of fertility support policies in China: A content and instrumental analysis
Source: PLoS One. 2025 Oct 9;20(10):e0332137. doi: 10.1371/journal.pone.0332137 (PMC12510515; doi:10.1371/journal.pone.0332137)
Supplement: S1 Appendix — (ZIP) [file pone.0332137.s001.zip › S1 Appendix. 226 original policy documents/92-机关事业单位工作人员带薪年休假实施办法(FBM-CLI-4-102180).doc]

机关事业单位工作人员带薪年休假实施办法

**发布部门：** [人事部(已撤销)](https://www.pkulaw.com/law/chl?Aggs.IssueDepartment=60211) [机构沿革](javascript:void(0))

**发文字号：**中华人民共和国人事部令第9号

**发布日期：**2008.02.15

**实施日期：**2008.02.15

**时效性：** [现行有效](https://www.pkulaw.com/law/chl?Aggs.TimelinessDic=01)

**效力级别：** [部门规章](https://www.pkulaw.com/law/chl?Aggs.EffectivenessDic=XE0301)

**法规类别：** [工资福利与劳动保险](https://www.pkulaw.com/law/chl?Aggs.Category=08707) [人事管理](https://www.pkulaw.com/law/chl?Aggs.Category=08804)

中华人民共和国人事部令
（第9号）

　　《机关事业单位工作人员带薪年休假实施办法》已经人事部部务会审议通过，现予发布，自公布之日起施行。

人事部部长　尹蔚民
二〇〇八年二月十五日

　　机关事业单位工作人员带薪年休假实施办法

　　**第一条**　为了规范机关、事业单位实施带薪年休假（以下简称年休假）制度，根据《[职工带薪年休假条例](https://www.pkulaw.com/chl/da3d4a4e60aa59d2bdfb.html)》（以下简称[《条例》](https://www.pkulaw.com/chl/da3d4a4e60aa59d2bdfb.html)）及国家有关规定，制定本办法。

　　**第二条**　[《条例》](https://www.pkulaw.com/chl/da3d4a4e60aa59d2bdfb.html)第[二条](https://www.pkulaw.com/chl/da3d4a4e60aa59d2bdfb.html#tiao_2)中所称“连续工作”的时间和第三条、第四条中所称“累计工作”的时间，机关、事业单位工作人员（以下简称工作人员）均按工作年限计算。

　　工作人员工作年限满1年、满10年、满20年后，从下月起享受相应的年休假天数。

　　**第三条**　国家规定的探亲假、婚丧假、产假的假期，不计入年休假的假期。

　　**第四条**　工作人员已享受当年的年休假，年内又出现[《条例》](https://www.pkulaw.com/chl/da3d4a4e60aa59d2bdfb.html)第[四条](https://www.pkulaw.com/chl/da3d4a4e60aa59d2bdfb.html#tiao_4)第（[二](https://www.pkulaw.com/chl/da3d4a4e60aa59d2bdfb.html#tiao_4_kuan_1_xiang_2)）、（[三](https://www.pkulaw.com/chl/da3d4a4e60aa59d2bdfb.html#tiao_4_kuan_1_xiang_3)）、（[四](https://www.pkulaw.com/chl/da3d4a4e60aa59d2bdfb.html#tiao_4_kuan_1_xiang_4)）、[（五）项](https://www.pkulaw.com/chl/da3d4a4e60aa59d2bdfb.html#tiao_4_kuan_1_xiang_5)规定的情形之一的，不享受下一年的年休假。

　　**第五条**　依法应享受寒暑假的工作人员，因工作需要未休寒暑假的，所在单位应当安排其休年休假；因工作需要休寒暑假天数少于年休假天数的，所在单位应当安排补足其年休假天数。

　　**第六条**　工作人员因承担野外地质勘查、野外测绘、远洋科学考察、极地科学考察以及其他特殊工作任务，所在单位不能在本年度安排其休年休假的，可以跨1个年度安排。

　　**第七条**　机关、事业单位因工作需要不安排工作人员休年休假，应当征求工作人员本人的意见。

　　机关、事业单位应当根据工作人员应休未休的年休假天数，对其支付年休假工资报酬。年休假工资报酬的支付标准是：每应休未休1天，按照本人应休年休假当年日工资收入的300%支付，其中包含工作人员正常工作期间的工资收入。

　　工作人员年休假工资报酬中，除正常工作期间工资收入外，其余部分应当由所在单位在下一年第一季度一次性支付，所需经费按现行经费渠道解决。实行工资统发的单位，应当纳入工资统发。

　　**第八条**　工作人员应休年休假当年日工资收入的计算办法是：本人全年工资收入除以全年计薪天数（261天）。

　　机关工作人员的全年工资收入，为本人全年应发的基本工资、国家规定的津贴补贴、年终一次性奖金之和；事业单位工作人员的全年工资收入，为本人全年应发的基本工资、国家规定的津贴补贴、绩效工资之和。其中，国家规定的津贴补贴不含根据住房、用车等制度改革向工作人员直接发放的货币补贴。

　　**第九条**　机关、事业单位已安排年休假，工作人员未休且有下列情形之一的，只享受正常工作期间的工资收入：

　　（一）因个人原因不休年休假的；

　　（二）请事假累计已超过本人应休年休假天数，但不足20天的。

　　**第十条**　机关、事业单位根据工作的具体情况，并考虑工作人员本人意愿，统筹安排，保证工作人员享受年休假。机关、事业单位应当加强年休假管理，严格考勤制度。

　　县级以上地方人民政府人事行政部门应当依据职权，主动对机关、事业单位执行年休假的情况进行监督检查。

　　**第十一条**　机关、事业单位不安排工作人员休年休假又不按本办法规定支付年休假工资报酬的，由县级以上地方人民政府人事行政部门责令限期改正。对逾期不改正的，除责令该单位支付年休假工资报酬外，单位还应当按照年休假工资报酬的数额向工作人员加付赔偿金。

　　对拒不支付年休假工资报酬、赔偿金的，属于机关和参照[公务员法](https://www.pkulaw.com/chl/823ca97b7f55d18cbdfb.html)管理的事业单位的，应当按照干部管理权限，对直接负责的主管人员以及其他直接责任人员依法给予处分，并责令支付；属于其他事业单位的，应当按照干部管理权限，对直接负责的主管人员以及其他直接责任人员依法给予处分，并由同级人事行政部门或工作人员本人申请人民法院强制执行。

　　**第十二条**　工作人员与所在单位因年休假发生的争议，依照国家有关公务员申诉控告和人事争议处理的规定处理。

　　**第十三条**　驻外使领馆工作人员、驻港澳地区内派人员以及机关、事业单位驻外非外交人员的年休假，按照[《条例》](https://www.pkulaw.com/chl/da3d4a4e60aa59d2bdfb.html)和本办法的规定执行。

　　按照国家规定经批准执行机关、事业单位工资收入分配制度的其他单位工作人员的年休假，参照[《条例》](https://www.pkulaw.com/chl/da3d4a4e60aa59d2bdfb.html)和本办法的规定执行。

　　**第十四条**　本办法自发布之日起施行。

© 北大法宝：（[www.pkulaw.com](https://www.pkulaw.com/)) 专业提供法律信息、法学知识和法律软件领域各类解决方案。北大法宝为您提供丰富的参考资料，正式引用法规条文时请与标准文本核对。
欢迎查看所有[产品和服务](https://www.pkulaw.com/helps/2.html)。法宝快讯：[如何快速找到您需要的检索结果？](https://www.pkulaw.com/helps/70.html)[法宝](https://www.pkulaw.com/helps/69.html)V6有何新特色？

扫描二维码阅读原文

**原文链接：**<https://www.pkulaw.com/chl/98ae806eba842966bdfb.html>
